# Supplementary material for: Pseudotime analysis reveals novel regulatory factors for multigenic onset and monogenic transition of odorant receptor expression
Source: Sci Rep. 2022 Sep 28;12:16183. doi: 10.1038/s41598-022-20106-w (PMC9519747; doi:10.1038/s41598-022-20106-w)
Supplement: Supplementary file 10 — Supplementary Information 10. [file 41598_2022_20106_MOESM10_ESM.pdf]

Pseudotime analysis reveals novel regulatory factors for multigenic onset and monogenic transition of odorant receptor expression

Mohammad Hussainy[1,2], **Sigrun I. Korsching**[2] **and Achim Tresch**[1,3,4]*
[1] Institute of Medical Statistics and Computational Biology, Faculty of Medicine, University of Cologne, Cologne, Germany
[2] Institute of Genetics, Faculty of Mathematics and Natural Sciences, University of Cologne, Cologne, Germany
[3] Cologne Excellence Cluster on Cellular Stress Responses in Aging-Associated Diseases (CECAD), University of Cologne, Cologne, Germany
[4] Center for Data and Simulation Science, University of Cologne, Cologne, Germany

* These authors contributed equally to this work: Sigrun I. Korsching (sigrun.korsching@uni-koeln.de); Achim Tresch (achim.tresch@uni-koeln.de).

Abstract

During their maturation from horizontal basal stem cells, olfactory sensory neurons (OSNs) are known to select exactly one out of hundreds of olfactory receptors (ORs) and express it on their surface, a process called monogenic selection. Monogenic expression is preceded by a multigenic phase during which several OR genes are expressed in a single OSN. Here, we perform pseudotime analysis of a single cell RNA-Seq dataset of murine olfactory epithelium to precisely align the multigenic and monogenic expression phases with the cell types occurring during OSN differentiation. In combination with motif analysis of OR gene cluster-associated enhancer regions, we identify known and novel transcription (co-)factors (Ebf1, Lhx2, Ldb1, Fos and Sspp2) and chromatin remodelers (Kdm1a, Eed and Zmynd8) associated with OR expression. The inferred temporal order of their activity suggests novel mechanisms contributing to multigenic OR expression and monogenic selection.

Single-cell RNA-seq datasets list

STable 2: List of single-cell RNA-seq datasets

Reference	Technology	Taxonomy	Tissue	Number of cells	GEO	Neuronal lineage
Nishijima et al., 2022	10X Genomics	Mus musculus	Olfactory Mucosa	>20K cells	GSE166612	>10K cells
Wang et al., 2022	10X Genomics	Mus musculus	MOE	>45K cells	GSE169011	>30K cells
van der Linden et al., 2020	10X Genomics	Mus musculus	Olfactory Epithelium		GSE157119	
Brann et al., 2020	10X Genomics	Mus musculus	Olfactory Epithelium	29585 cells	GSE153730	17666 mOSNs and 4674 iOSNs
Gadye et al., 2017	SMART Technology	Mus musculus	Olfactory Epithelium	1384 cells	GSE99251	119 cells
Lin et al., 2017	SMART Technology	Mus musculus	Olfactory Epithelium and Neurog1-eGFP+ progenitors	469 cells	GSE92842	
Fletcher et al., 2017	SMART Technology	Mus musculus	Olfactory Epithelium	849 cells	GSE95601	~294 cells
Hanchate et al 2015	SMART Technology	Mus musculus	Olfactory Epithelium	93 cells	GSE75413	93 cells
Tan et al., 2015	SMART Technology	Mus musculus	Olfactory Epithelium	178 cells	SRR2911286	
Oliva et al., 2022	10X Genomics	Homo sapiens	Olfactory Epithelium	36091 cells		
Durante et al., 2020	10X Genomics	Homo sapiens	Olfactory Epithelium	>28K cells	GSE139522	~694 cells

Distribution of detected genes per cell before and after imputation

SFigure 1a: Library size per cell after final filtration and without imputation.
A) Violin plot of number of expressed genes per cell in each cell type. **B)** Violin plot of number of reads (nCounts) per cell in each cell type where each black dot represents the library size of a single cell.

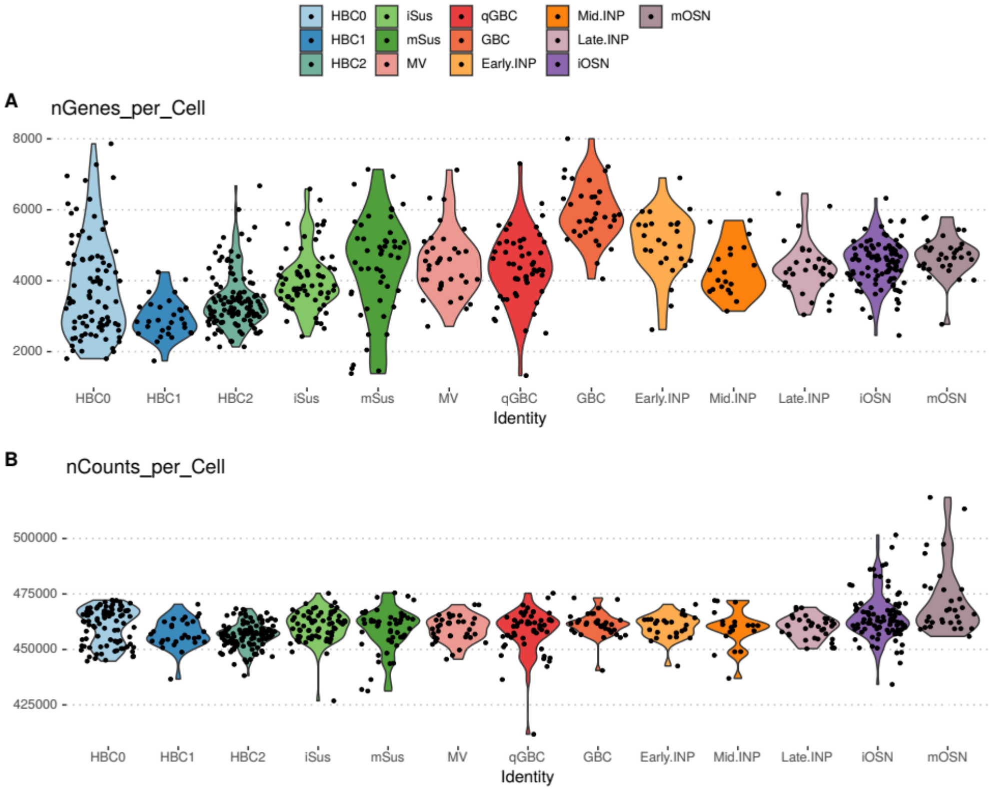

SFigure 1b: Distribution of genes per cell after imputation (dealing with dropouts).

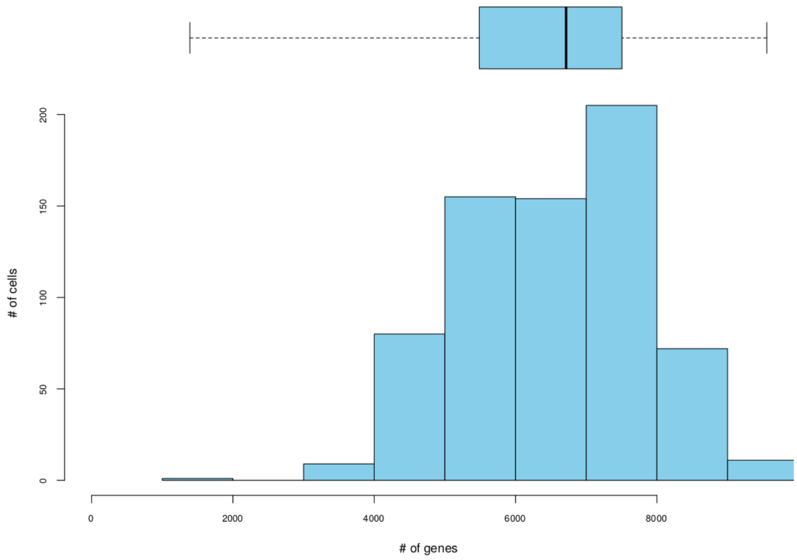

Cell type marker genes

STable 2: List of marker genes that we used to assign individual cells to cell types, according to their scRNA-Seq expression profile.

**Cell type**	**Marker gene(s)**	**References**
Horizontal basal stem cells (HBC0, HBC1 and HBC2)	*Trp63, Krt5* and *Krt14*	Fletcher et al. 2017; Fletcher et al. 2011
Sustentacular cells<br><br>(iSus and mSus)	*Cyp2g1, Notch2, Hey1, Sox2* and *Cyp1a2*	Rodriguez et al. 2008; Fletcher et al. 2017; Durante et al. 2020
Microvilious cells (MV)	*Ascl3, Cftr* and *Coch*	Fletcher et al. 2017; Genovese & Tizzano 2018; Durante et al. 2020
Globosal basal stem cells (qGBC and GBC)	*Lgr5, Tmprss4, Kit Mki67 and Top2a, Hes6* and *Ascl1*	Leung et al. 2018; Goss et al. 2016; Packard et al. 2016; Goldstein et al. 2015; Jang et al. 2014; Chen et al. 2014; Huard et al. 1998; Caggiano et al. 1994
Immediate neuronal precursor 1 and 2<br><br>(INP.Early and INP.Mid)	*Neurod1, Neurog, Top2a, Mki67 and Lhx2*	Fletcher et al. 2017; Packard et al. 2016
Immediate neuronal precursor 3<br><br>(INP.Late)	*Lhx2, Ebf1 and Gap43*	Fletcher et al. 2017; Packard et al. 2016
Immature olfactory sensory neurons (iOSN)	*Gng8, Trib3 and Gap43*	Hanchate et al. 2015;Lin et al. 2017; Fletcher et al. 2017; Durante et al. 2020
Mature olfactory sensory neurons (mOSN)	*Gng13, Gnal and OMP*	Hanchate et al. 2015;Lin et al. 2017;Fletcher et al. 2017;Durante et al. 2020

DE Plots

SFigure 2a: Heatmap plot shows the expression profiles of top 10 differentially expressed genes in each cell type starting from HBC0 (top left) and ending with mOSN (bottom right). The gradient scale colors represent the normalized expression in log scale: white (no expression), blue (low to high expression) and red color (very high expression).

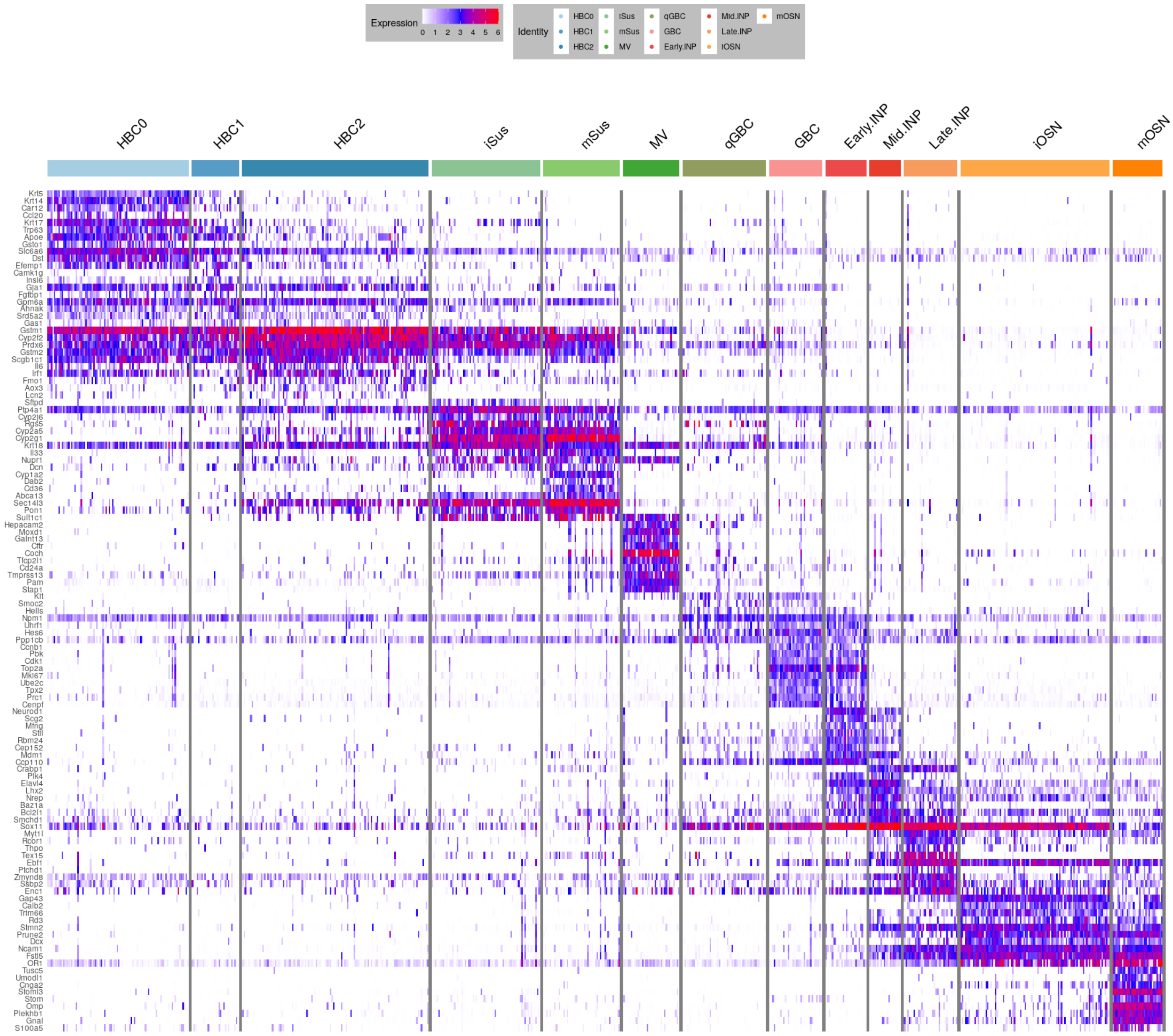

SFigure 2b: The upregulated and downregulated (co-)TF genes from stage to stage along neuronal lineage. We selected (co-)TFs with an adjusted p-value less than 0.05 (y-axis, $\log_{10}$ Bonferroni adjusted p-value) and an average expression change of at least 2 fold (x.axis, $\log_2$ fold).

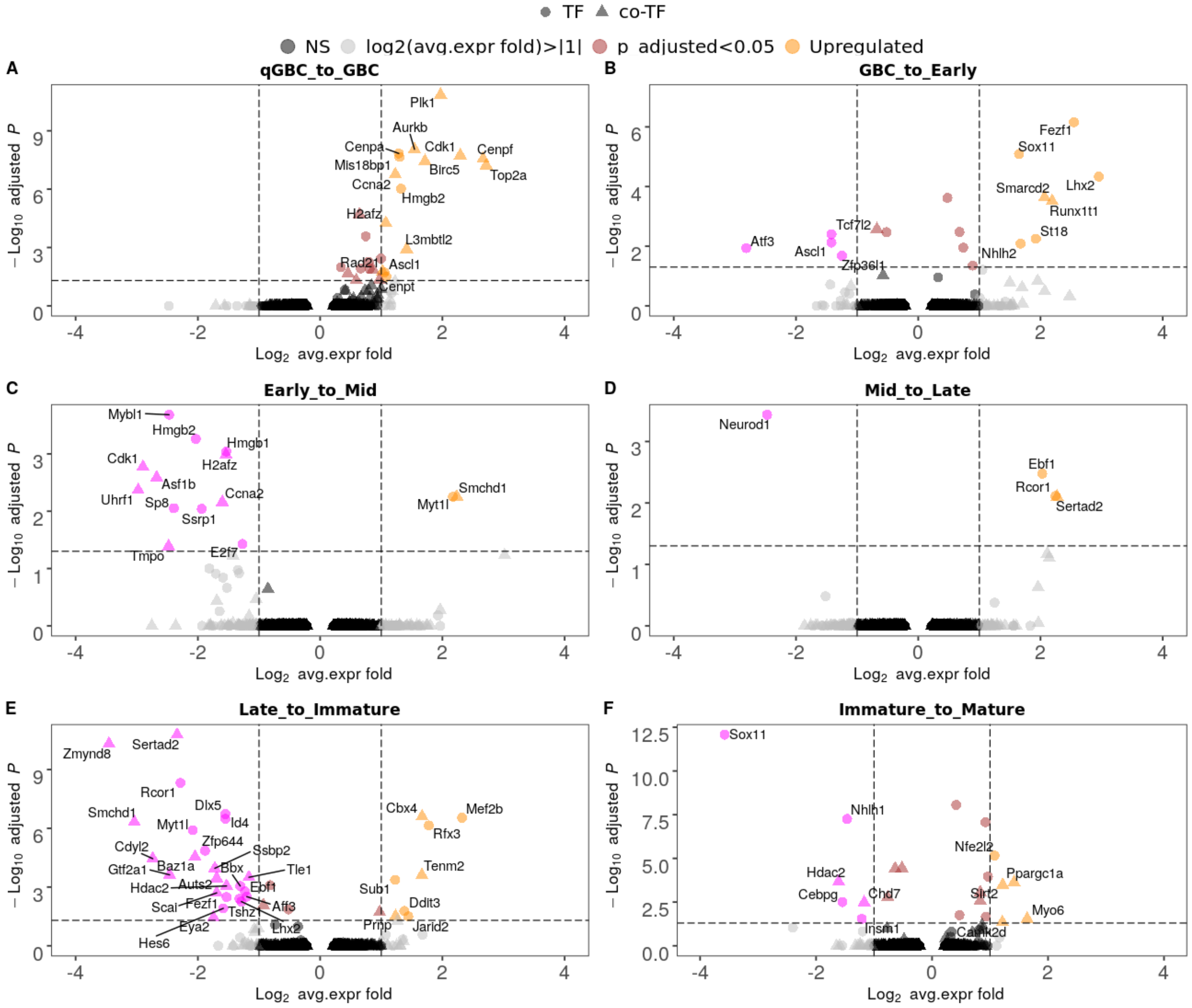

10x Genomics dataset

SFigure 3a: Library size per cell of 10x genomics dataset obtained from Wang et al., 2022. The main cell types of MOE (HBC, SUS, MV, GBC, INP, iOSN and OSN) were selected to visualize library size of 10x genomics dataset. The total number of cells for each cell type is shown on the top of corresponding cell type.

A) Violin plot of number of expressed genes per cell in each cell type. **B)** Violin plot of number of reads (nCounts) per cell in each cell type where each black dot represents the library size of a single cell.

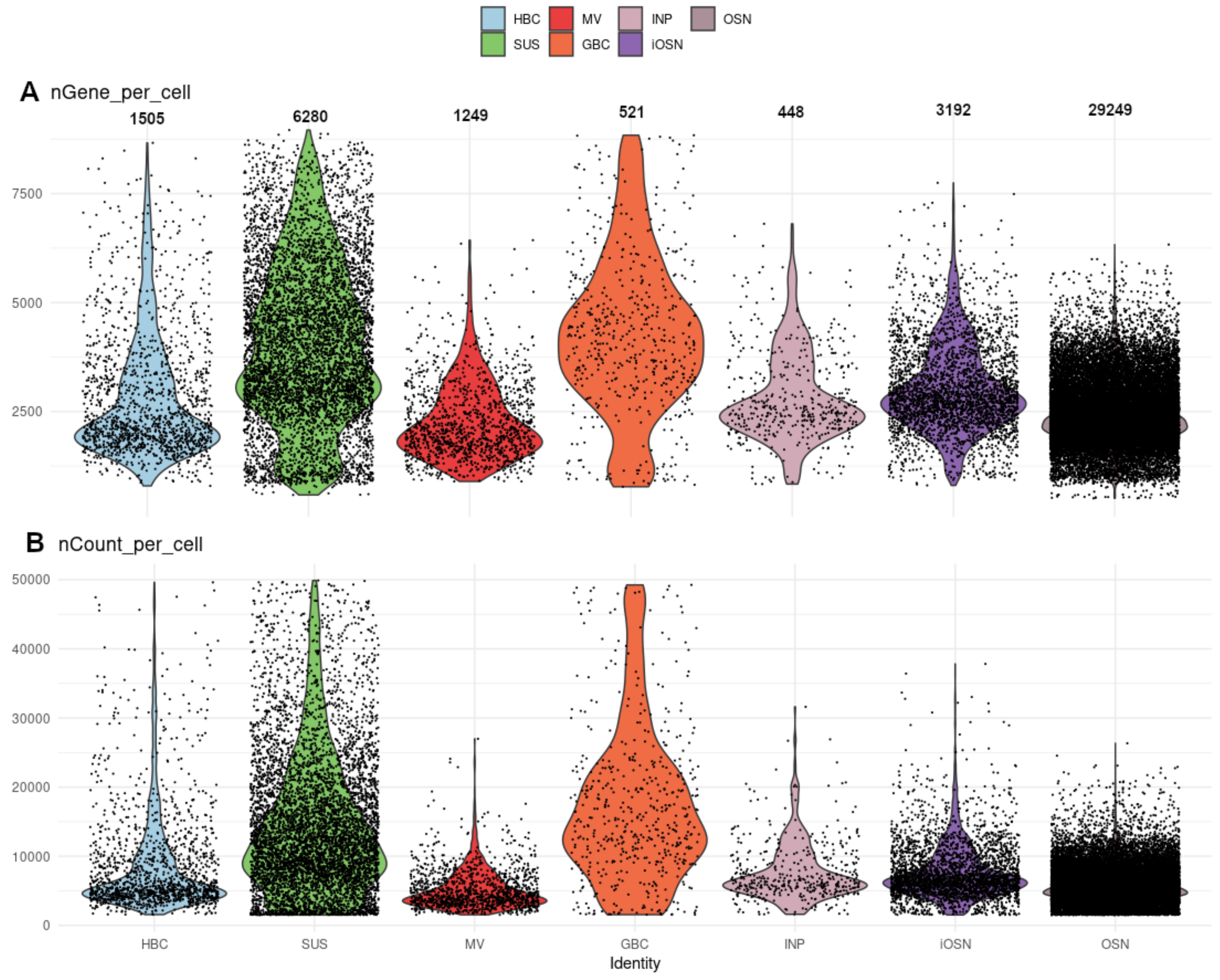

SFigure 3b: UMAP plot of re-clustering result of neuronal lineage cells. Left panel shows the re-clustering result, and right panel shows the clusters as obtained from Wang et al.,2022.

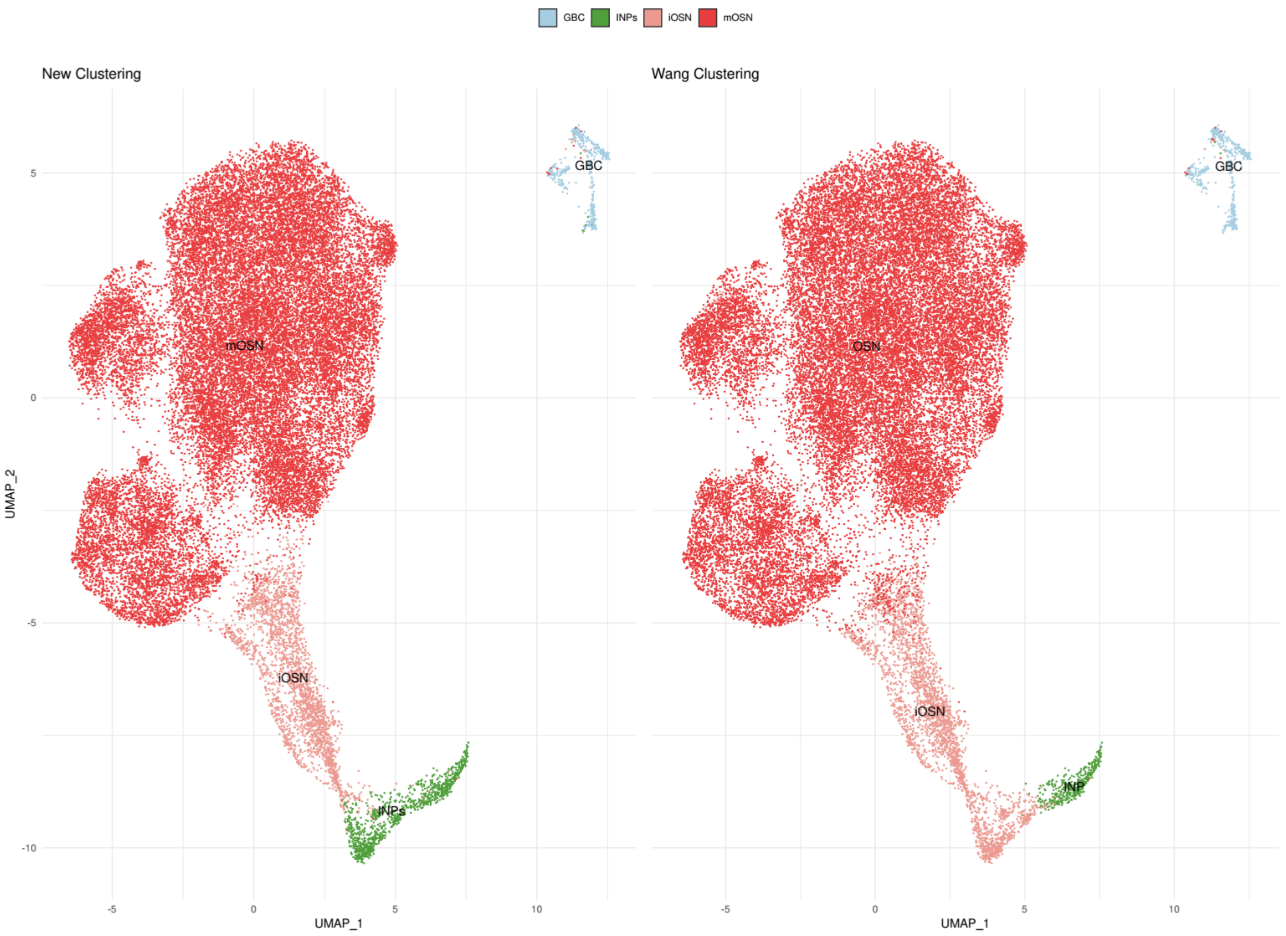

Comparison between SMART-Seq2 and 10x Genomics

SFigure 4a: Comparison between the expression profiles of the winner and runners up of ORs.

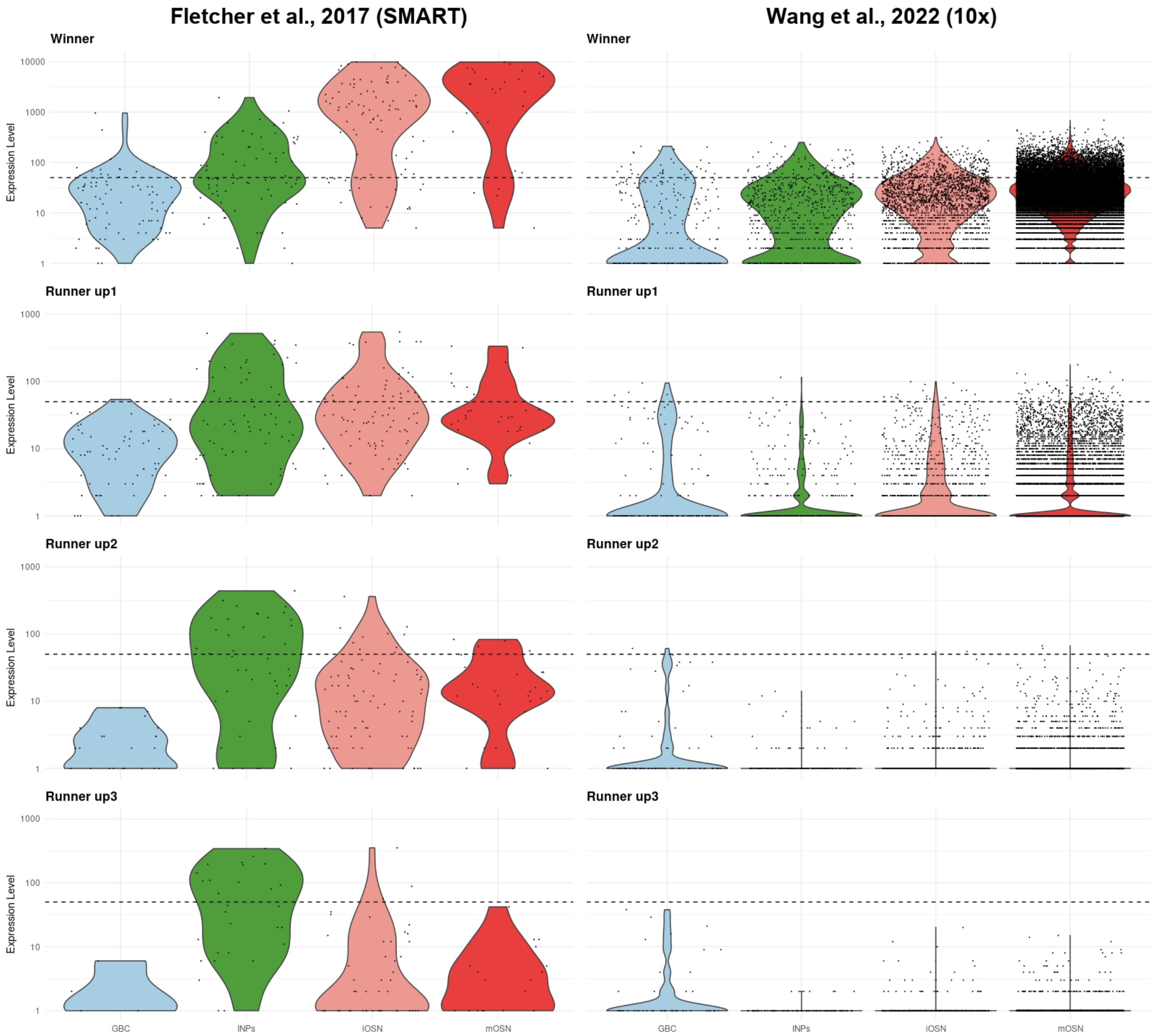

SFigure 4b: Comparison between smart-seq2 and 10x genomics dataset shows the expression profiles of predicted transcription factors (TFs) and chromatin remodelers (CRs) shown on Figure 4b. **A,B) shows the expression profiles of TFs.** The first violin plot row of each profile of the three groups is from smart-seq2 and the second one is from 10x genomics. **C,D) shows the expression profiles of CRs.** The first violin plot row of each profile of the three groups is from smart-seq2 and the second one is from 10x genomics.

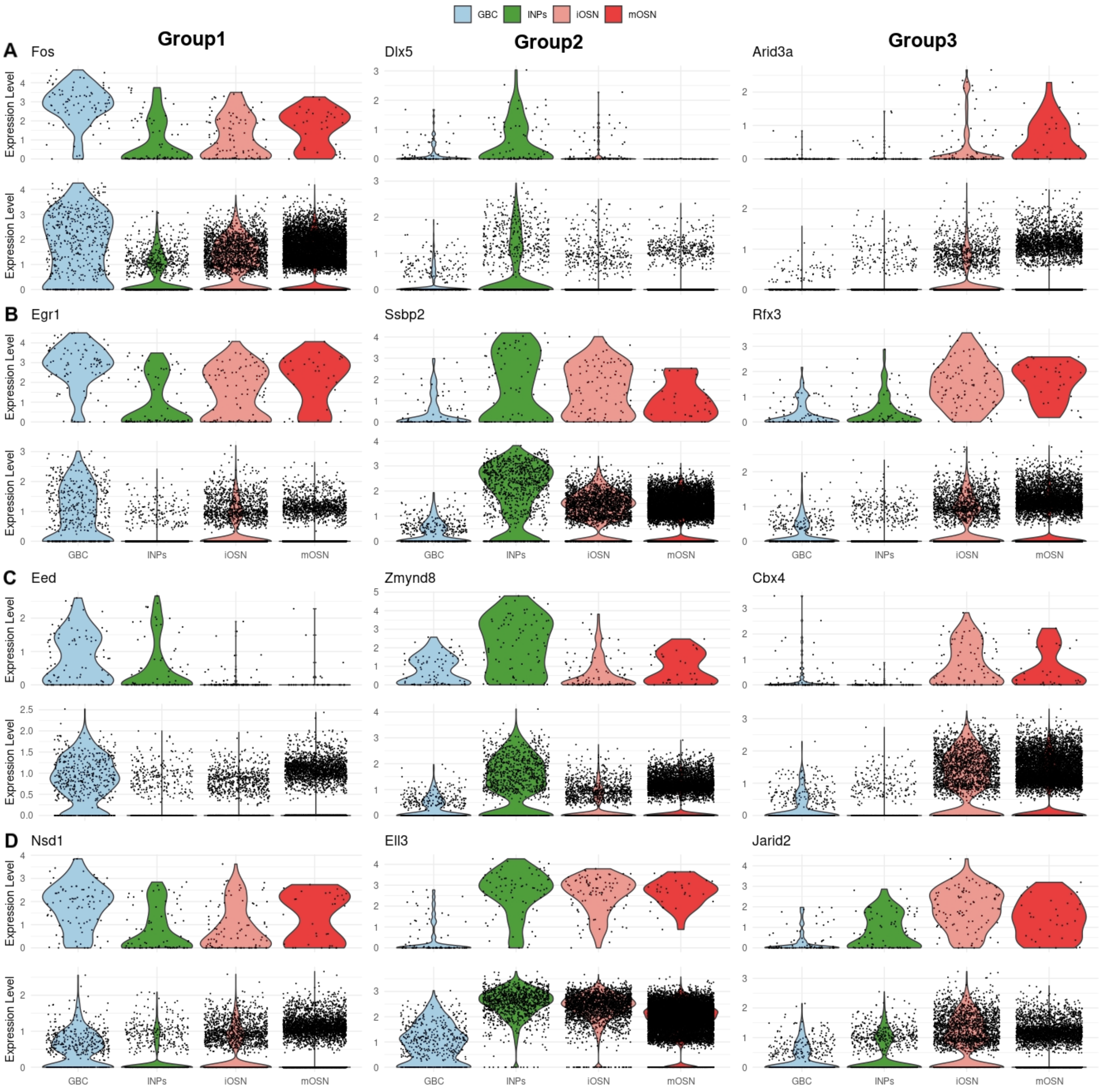

Coexpression analysis of olfactory receptor genes

SFigure 5: Coexpression analysis of olfactory receptor genes related to the same cluster. We performed MCMC sampling to assess whether the observed average number of OR gene clusters co-expressing at least 2 OR genes is in the expected range. We first construct a binary cells x genes matrix indicating whether or not a gene is expressed in a cell. From this matrix, we compute as a test statistic the relative frequency of clusters expressing at least 2 genes among all clusters. The same is done for a uniform random sample of all binary matrices that have the same marginal count frequencies as the original matrix. The random samples are drawn by the curveball algorithm (see SCode 1 below). The figure shows the histogram of the test statistic for this random sample. The red dotted line indicates the actually observed value, corresponding to a (two-sided) p-value of 0.112.

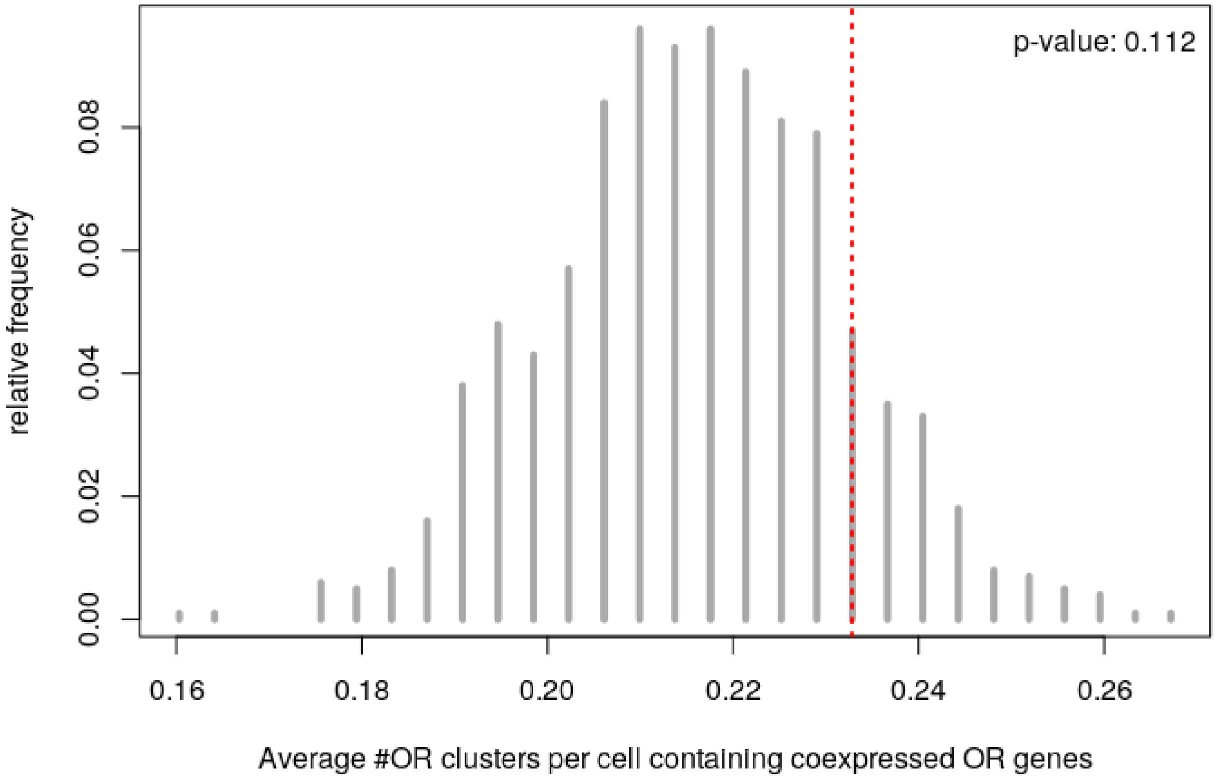

SCode 1: Coexpression analysis of olfactory receptor genes

We perform MCMC sampling to draw samples uniformly from the set of binary matrices with fixed marginals. We use the 'Curveball' algorithm of Strona et al., Nat.Comm 2014, https://doi.org/10.1038/ncomms5114.

```
# This is the R code provided in Strona et al., Supplementary Software 5

curve_ball<-function(m){

  RC=dim(m); R=RC[1]; C=RC[2]; hp=list()

  for (row in 1:dim(m)[1]) {hp[[row]]=(which(m[row,]==1))}

  l_hp=length(hp)

  for (rep in 1:(5*l_hp)){

    AB=sample(1:l_hp,2); a=hp[[AB[1]]]; b=hp[[AB[2]]]; ab=intersect(a,b)

    l_ab=length(ab); l_a=length(a); l_b=length(b)

    if ((l_ab %in% c(l_a,l_b))==F){

      tot=setdiff(c(a,b),ab); l_tot=length(tot)

      tot=sample(tot, l_tot, replace = FALSE, prob = NULL)

      L=l_a-l_ab; hp[[AB[1]]] = c(ab,tot[1:L])

      hp[[AB[2]]] = c(ab,tot[(L+1):l_tot])}

  }

  rm=matrix(0,R,C)

  for (row in 1:R){rm[row,hp[[row]]]=1}

  rm

}
```

Let expression_mat the original (binarized) genes x cells expression matrix. It contains a 1 whenever the gene is detected in a cell with at least one count. Let assignment_mat the (binary) OR clusters x genes incidence matrix. It contains a 1 at position (c,g) exactly if a gene g is localized in cluster c. Both matrices are available in the Supplementary Materials (Excel 2).

For each MCMC sample, we record the number of OR genes (co-)expressed in each OR cluster.

```
MCMC_samples = 10^3 # number of MCMC samples we want to draw

MCMC_stats = matrix(0,nrow=nrow(assignment_mat),ncol=MCMC_samples)
```

```
# MCMC_stats contains the number of times an OR cluster had >=2 coexpressed genes (across all cells), for each OR cluster
mat = expression_mat
# assignment_mat %*% mat is a OR clusters x cells matrix
# containing the #active OR genes (in a given cluster and a given cell)
# MCMC_stats[,j] contains, for each OR cluster the #cells in which
# coexpression  found for this cluster.
MCMC_stats[,1] = rowSums((assignment_mat %*% mat) > 1)
# The original expression matrix is counted as the first sample
cat("Progress: ")
for (j in 2:MCMC_samples){
  mat = curve_ball(mat)
  MCMC_stats[,j] = rowSums((assignment_mat %*% mat) > 1)
  if(j %% (MCMC_samples %/% 25) == 0) cat(".")
}
cat("\n")
```

The test statistic is the relative frequency (in the population of all cells) by which an OR cluster has more than 1 OR gene expressed.

```
cells = ncol(expression_mat)
coexpressed = colSums(MCMC_stats) / cells
# the relative frequency at which we observe coexpressed genes in a cluster
# (across all clusters, per cell), for each MCMC step
original_stats = sum((assignment_mat %*% expression_mat) > 1) / cells
null_distr = table(coexpressed)
location = as.numeric(names(null_distr))
probabilities = as.numeric(null_distr)
probabilities = probabilities/sum(probabilities)
plot(location,probabilities,type="h",lwd=4,col="dark grey",
     xlab="Average #OR clusters per cell containing coexpressed OR genes",
```

```
     ylab="relative frequency")

abline(v=original_stats,col="red",lwd=2,lty=3)


larger = which(location >= original_stats)

pvalue = sum(probabilities[larger])

legend("topright",paste("p-value:",signif(pvalue,digits=3)),bty="n")
```

Genomic locations of OR clusters and Greek islands

SFigure 6: Genomic locations of OR gene clusters (black bars) and Greek island enhancer regions (red bar) in each murine chromosome. Chromosomes are scaled to identical width for visualization purposes.

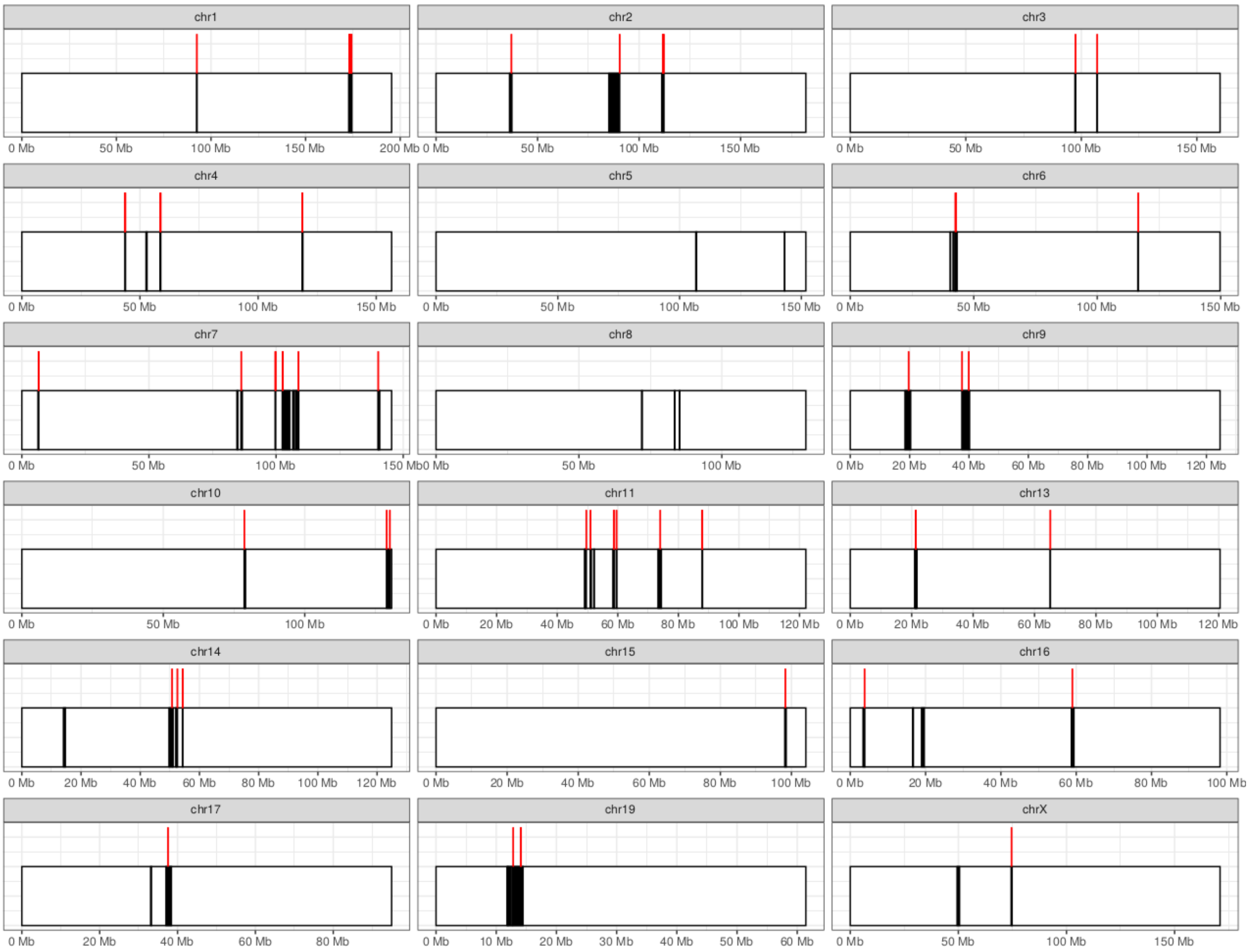

VlnPlot of all TFs found in motif search

SFigure 7: (co)-TFs binding into Greek islands (Motif search results) that express at least 35 counts in at least 15 out of a total of 294 cells.

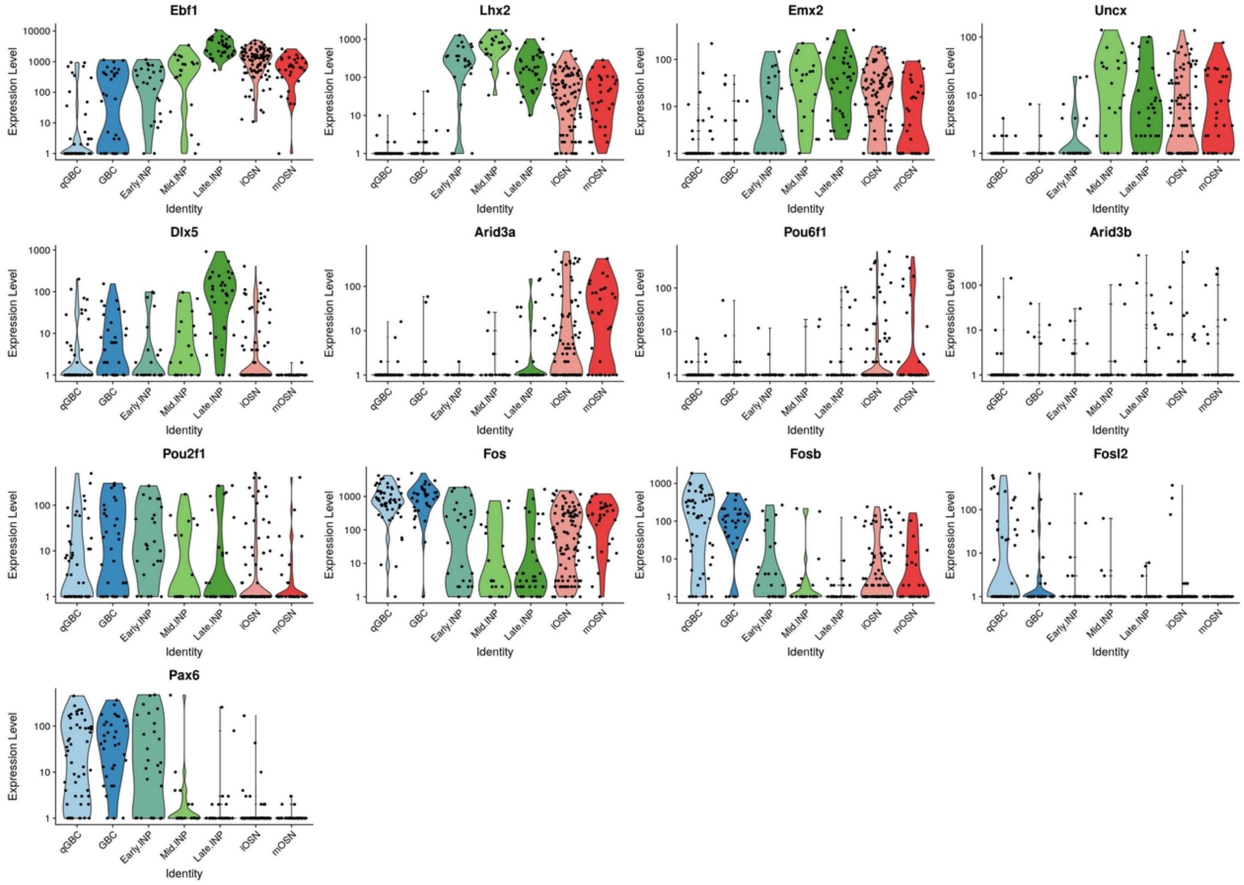

Pseudotime expression profiles for other factors

SFigure 8: Other important transcription factors (TFs) and chromatin remodelers (CRs) associated with the three phases of OR expression. **A)** TFs and co-TFs. **B)** CRs

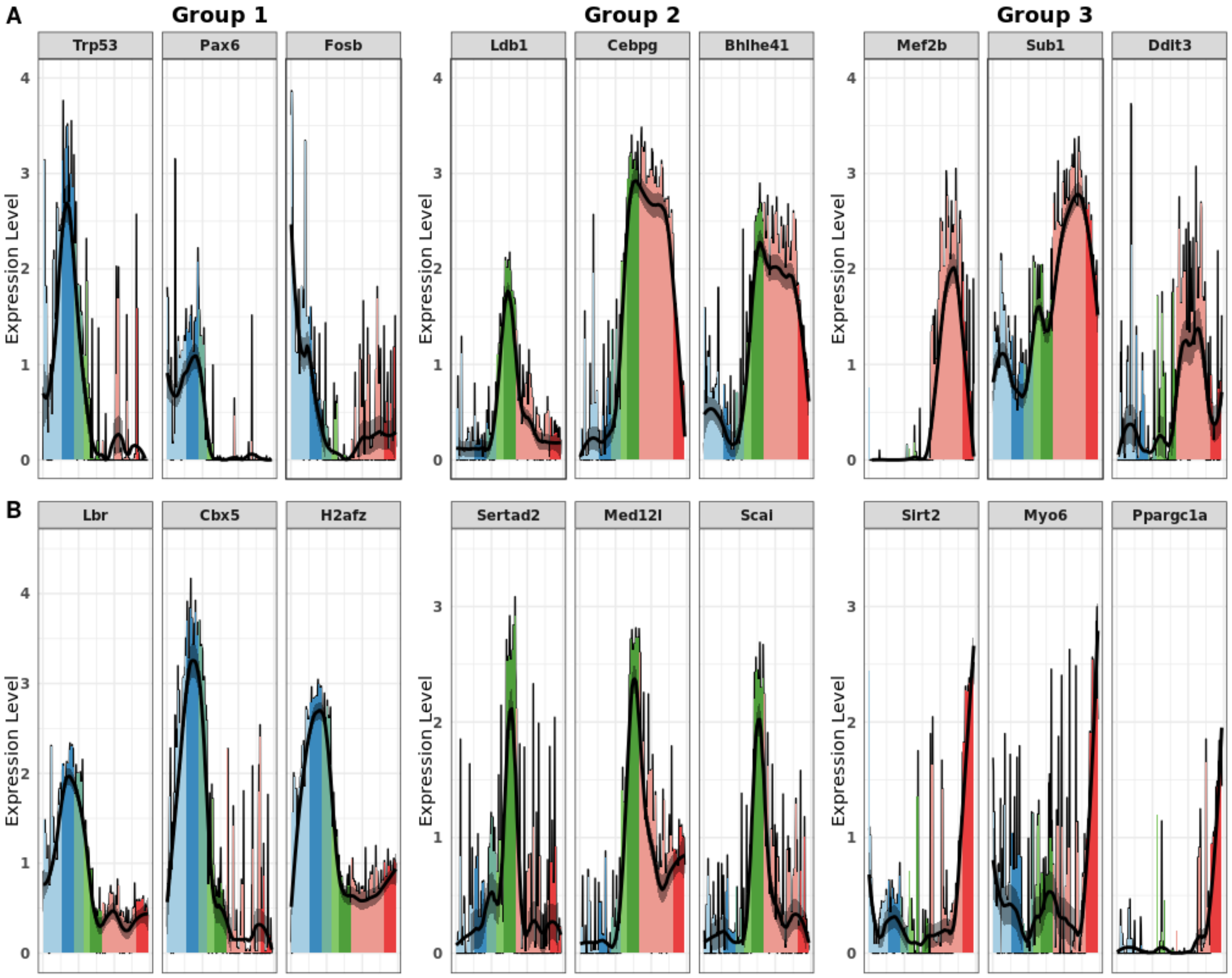

PRC2 complex (H3K27 methylation)

SFigure 9: **Transcriptional dynamics of PRC2 complex.** The top row shows the pseudotime expression timecourse for each of the PRC2 subunits (Eed, Ezh2, Suz12) separately. The bottom row shows the expression distribution of each subunit per cell type. Numbers indicate the p-value (Wilcoxon rank sum test) of cell type transitions that show a significant expression change. Note that the expression of the Eed subunit is almost zero in Late.INP stage, indicating that the elimination of PRC2 is done during transition from Mid to Late.INP stage.

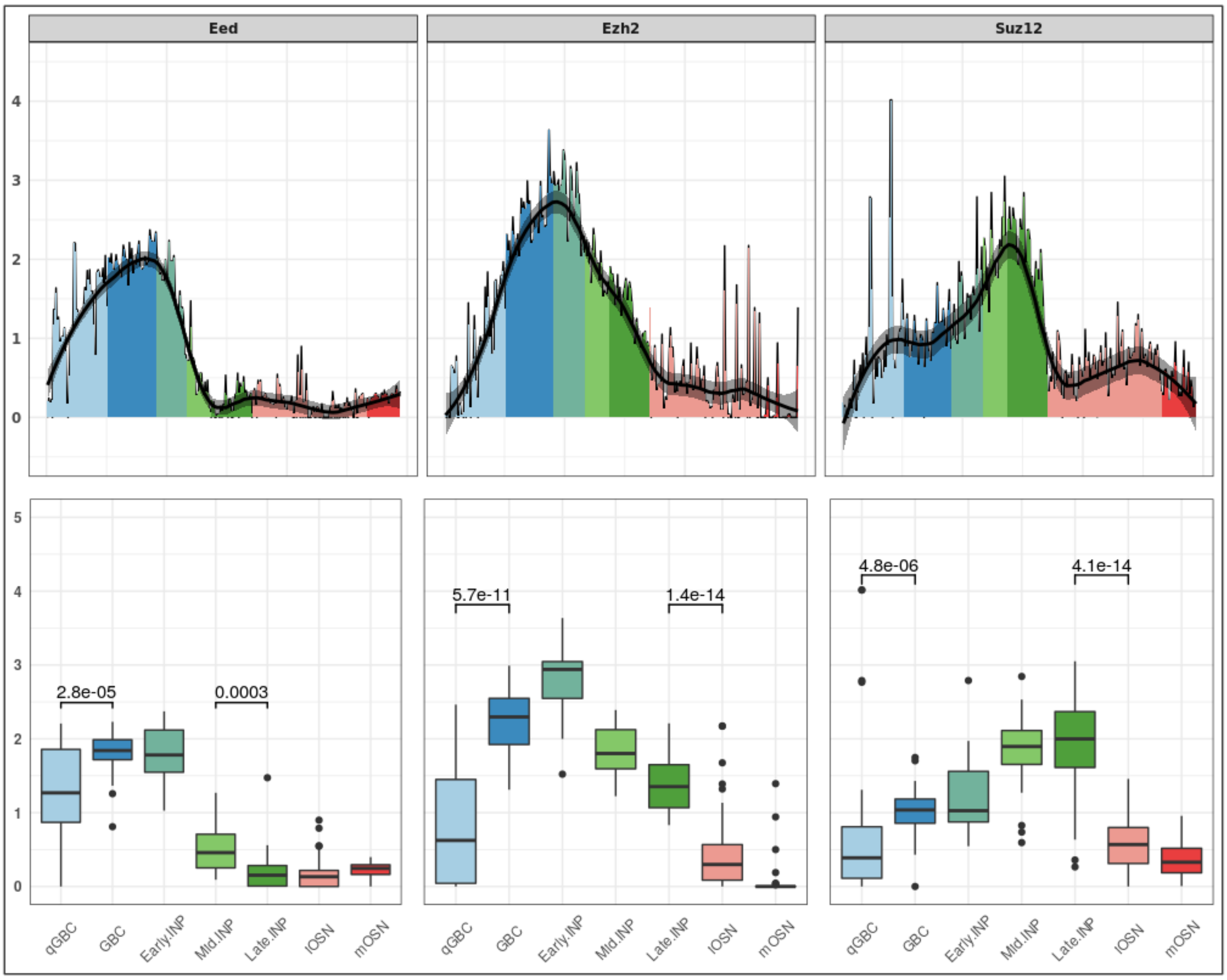

Pseudotime expression profiles for other factors

SFigure 10: A) pseudotime expression profiles of Kdm1a and factors that play a role in changing the function of Kdm1a. B) Feedback Signal. Translation of the newly transcribed OR mRNA activates a co-opted arm of the unfolded protein response (Dalton et al., 2013) and induces a feedback signal (Lewcock and Reed, 2004; Serizawa et al., 2005; Shykind et al., 2004). Atf5 and Adcy3 are proposed to be involved in this process where Atf5 promotes Adcy3 expression which in turn downregulates Kdm1a (LSD1) preventing the de-silencing of another OR gene (Dalton et al., 2013; Lyons et al., 2013).

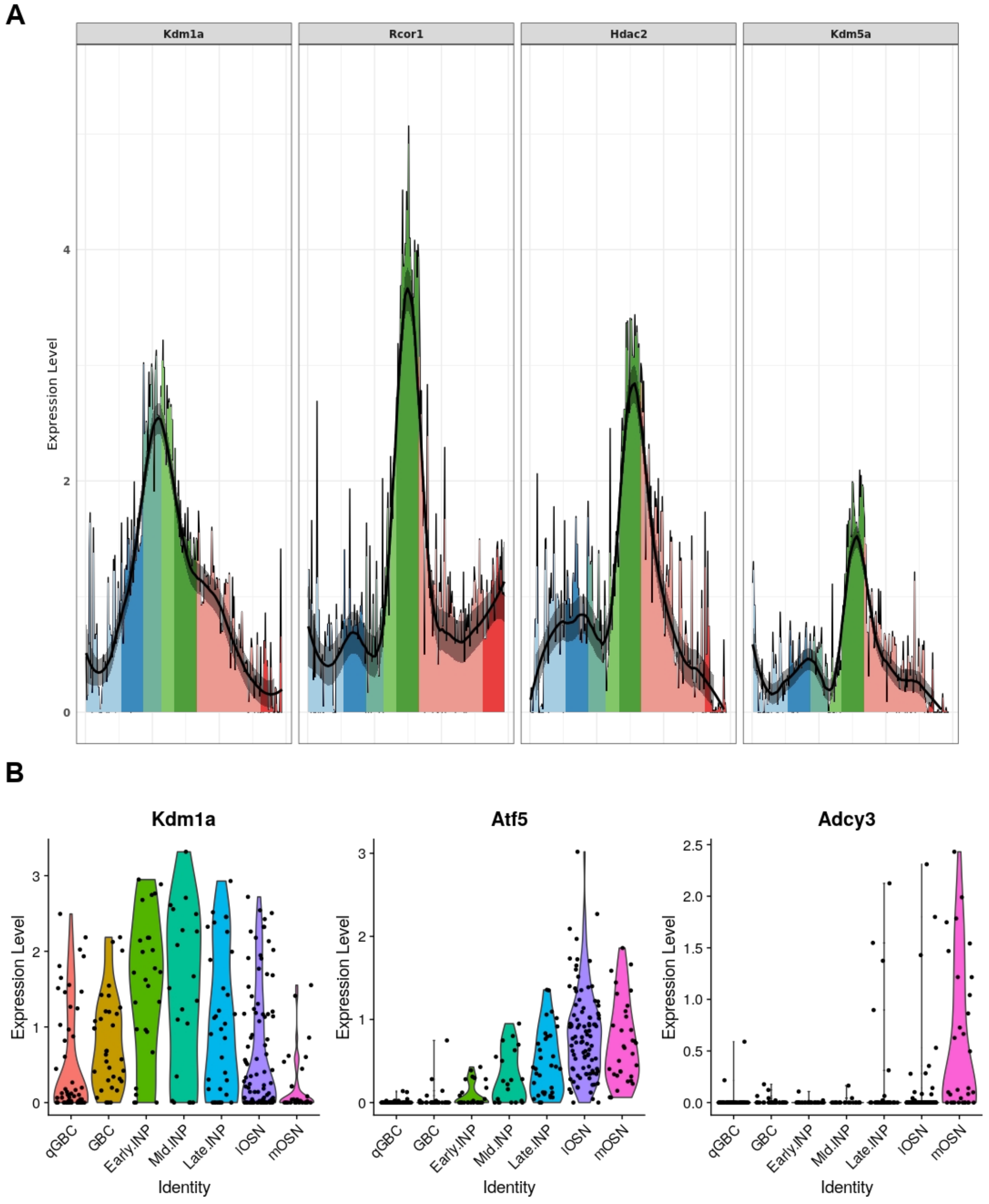

Monoallelic factors

SFigure 11: Monoallelic silencing factors. The transcriptional dynamics of Smchd1 (light blue) and Cdyl2 (blue) show the same expression pattern along neuronal lineage, indicating that they probably are recruited for the same function (stabilizing the monoallelic selection after asynchronous replication of OR genes) during their peak period of neuronal lineage (Mid and Late.INP stages).

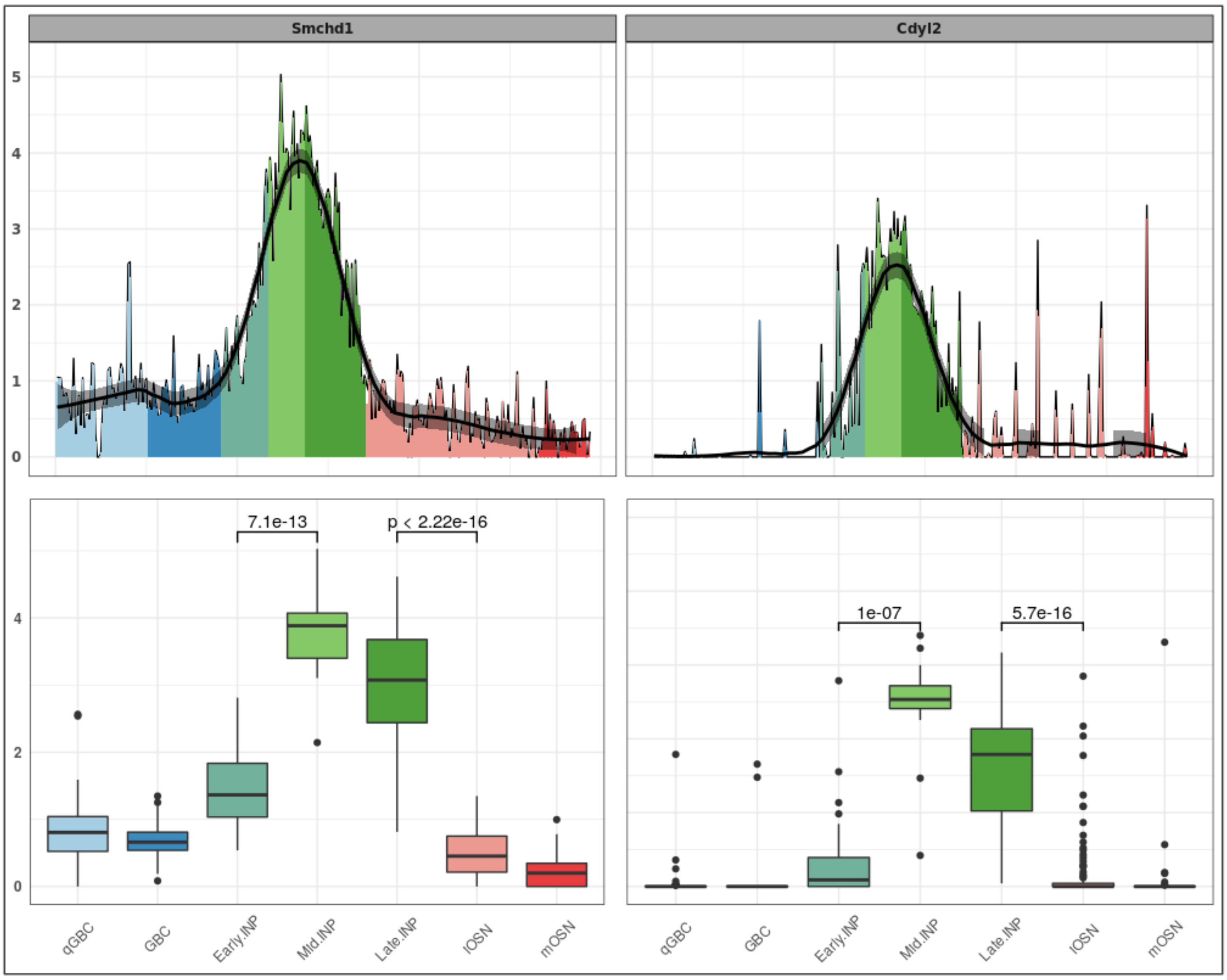

Supplementary Files

Excel file 1

Excel file 2

Excel file 3

Excel file 4

Excel file 5,6

Excel file 7

Greek_islands_Sequences.fasta

modified_filtering.R
__exclude.Rdata
